# Pamphlets and Papers Received

**Published:** 1882-05-20

**Authors:** 


### *PAMPHLETS AND PAPERS RECEIVED.*

WORKING BULLETIN, for the Scientific Investigation of Drugs. We are in receipt of copies of the above from the Scientific Department of the energetic estalishment of Parke, Davis & Co., Detroit. The Working Bulletin is distributed gratuitously to Colleges, Universities, Hospitals, etc., and samples and preparations of the drugs investigated, also furnished.

The object is to promote investigation in the science of drugs, and by publishing results, to place upon record useful and practical information.

The botanical history, the cultivation, structure and chemical composition of drugs are given, together with the doses and medical properties as drawn from reports of cases, etc. At the end of each year, the several reports issued, will be compiled into an annual. This is certainly a great, liberal and useful work, and furnishes another evidence of the indomitable energy and enterprise of the house of Parke, Davis & Co.

THE Cornell University Register, 1881-1882, Ithaca, New York, containing catalogue, curriculum, etc., etc.

RELIGIO-PHILOSPHICAL JOURNAL—Devoted to Spiritual Philosophy. The most ably edited and reliable of its class. Jno. C. Bundy editor and publisher, Chicago, Ill.

ON some points in connection with the treatment of Sterility, by A. Reeves Jackson, A.M., M.D., Chicago. Ill.

A FEW remarks upon Fellows' Hypophosphites of Quinine, Strychnine, Iron, Lime, Potassa and Manganese. For the Medical Profession. London: Jas. I. Fellows, Snow Hill, E. C., 1881.

THE Student's Manual of Venerial Diseases, being a concise description of those affections and of their treatment. By Berkley Hill, Prof. of Clinical Surgery in University College, London; Surgeon to the University College and to the Lock Hospitals; and by Arthur Cooper, late House Surgeon to the Lock Hospital. Second edition. New York: William Wood & Co. 1881.

CURRENT Fallacies about Vaccination. A letter to Dr. W. B. Carpenter, C.B., etc., etc., by P. A. Taylor, M. P. Second edition of 100,000; with additional remarks on Dr. Carpenter's article on Disease-germs, in the Nineteenth Century Magazine for October.

OBSERVATIONS on Surgery in Children, by Edward Borck, M.D., St. Louis, Mo., Prof. of Surgical Diseases of Children and Abdominal and Clinical Surgery in the "College for Medical Practitioners," St. Louis, etc. Read before the St. Louis Medical Society, April 1st, 1882.

